# Exploring perspectives and boundaries in neurosurgical career pathways for generation Z in German-speaking countries

**DOI:** 10.1016/j.bas.2025.104382

**Published:** 2025-08-06

**Authors:** S. Motov, M.N. Stienen, F.C. Stengel, M. Schwake, P. Schuss, S. Ridwan

**Affiliations:** a Department of Neurosurgery, Spine Center HOCH Health Ostschweiz, Cantonal Hospital St. Gallen, Switzerland; b Department of Neurosurgery, University Hospital Münster, Münster, Germany; c Department of Neurosurgery, BG Klinikum Unfallkrankenhaus Berlin, Germany; d Department of Neurosurgery, Klinikum Ibbenbueren, Ibbenbueren, Germany

**Keywords:** Generation Z, Neurosurgery, Career choice, Medical education, Work-life balance, Social media

## Abstract

**Objective:**

Generation Z (born 1997–2010) is the first generation to grow up entirely in the digital age. This study investigates how this generation perceives neurosurgery as a career path in German-speaking countries.

**Research question:**

What are the motivations, barriers, and expectations of Generation Z regarding a career in neurosurgery, and how do these differ between medical students and residents?

**Methods:**

A 20-item online survey (including Likert scales, single-choice, and numeric rating scales) was distributed via professional and student associations in Germany and Switzerland between February 9 and March 30, 2025. Participants included medical students and residents. Data were analyzed using Stata 18.0.

**Results:**

A total of 351 responses were analyzed (65 % students, 35 % residents; mean age 25 years; 58 % female). Interest in neurosurgery was significantly higher among residents (80 %) than students (52 %, *p* < 0.001), especially in clinical semesters (57 % vs. 36 %, *p* = 0.006). The average likelihood of pursuing neurosurgery was 70/100, higher among residents (90 vs. 56, *p* < 0.001). Key motivations included fascination with surgery (students: 58 %, residents: 62 %), scientific interest, and clinical variety. Deterrents were a lack of mentorship (13 % vs. 24 %) and rigid hierarchies. Students prioritized flexibility (37 %), while residents favored more hands-on training (35 %, *p* = 0.002). Work-life balance was a major concern for both groups (≥74 %). Structured mentorship was important to 88 % (*p* = 0.024).

**Conclusion:**

Generation Z demonstrates above-average interest in neurosurgery but emphasizes the need for better mentorship, work-life balance, and training reforms. Tailored educational strategies and modernized work models may enhance recruitment and long-term engagement in neurosurgical careers.

## Highlights

• Above-average interest in neurosurgery among Generation Z.
• Key deterrents include long working hours, hierarchical structures, and a lack of mentorship.
• Work-life balance and flexibility are priorities for Generation Z.
• Social media is underutilized.

## 1 Introduction

Generation Z, born between 1997 and 2010, is the first generation to have grown up entirely within a digitally interconnected world defined by ubiquitous Internet access, smartphones, and social media (SoMe). Often characterized as tech-savvy, interconnected, and adept at multitasking, this generation also places strong value on personalized mentorship, psychologically safe work environments, work-life balance, and mental well-being (Mavrogenis et al., 2024).

Despite the appeal of neurosurgery – an innovative specialty that integrates cutting-edge technology and offers a wide range of subspecialties – recent years have seen a decline in interest among potential medical trainees (Bandiera et al., 2014). Several factors have been previously identified as deterrents to entering the field: the extended duration of training, perceived lifestyle changes, limited early exposure to neurosurgery, and scarcity of accessible mentors(Stienen et al., 2016a, 2016b).

As neurosurgical professionals actively engaged in educational and outreach activities such as webinars, podcasts, and workshops, we sought to better understand how the aspirations and expectations of Generation Z align with a career in neurosurgery. This study investigates the career motivations, perceived barriers, and future expectations among medical students and neurosurgical residents in Germany and Switzerland. Our primary goal is to identify actionable strategies that can effectively support, engage, and inspire the next generation of neurosurgeons.

## 2 Methods

### 2.1 Web-based survey and distribution

A 20-question online questionnaire (Likert scale, single best choice, and 0–100 scale questions) in German language was designed using the SurveyMonkey platform (https://www.surveymonkey.com) and distributed between the 9th of February and the March 30, 2025 b y the DGNC, Connectome, NCH TO GO, and BVMD section email platforms. The survey targeted medical students as potential neurosurgical prospects in general and current neurosurgical residents in training. Thus, the questionnaire was distributed via newsletter and online bulletin boards of medical student councils in Germany and through the YSSA (Young Surgical Students Association) in Switzerland to reach as many students as possible, possibly independent of their interest in a neurosurgical career. Students with an interest in neuroscience were reached additionally via a national students’ association for neurosurgery, neurology, and neuroscience (Connectome). The survey was also distributed among neurosurgical residents and students by the German Neurosurgical Society (DGNC), the online platform for continuing medical education Neurosurgery To Go (NCH TO GO; www.nchtogo.de) using online newsletter. We also encouraged personal and social media promotion among participants.

### 2.2 Survey design

The questionnaire was structured in three distinct sections. The first part captured demographic information and general characteristics, while the second part centered on career motivations and deterrents specific to neurosurgical training. The last part focused on the utilization of social media (SoMe) and emerging technologies (ET) in professional context. The complete survey instrument can be found in **Supplemental Nr. 1**. Survey results were checked for duplicates and missing data.

### 2.3 Statistical analysis

All statistical analyses and generation of all graphs were performed using StataSE 18.0 (StataCorp. 2023. Stata Statistical Software: Release 18. College Station, TX: StataCorp LLC). Descriptive statistics were reported as frequencies (%) for categorical variables or means with standard deviation (SD) for continuous variables. Chi-square and Shapiro-Wilk tests were applied to compare categorical and distributional differences between groups (students/residents, preclinical (<3rd year)/clinical (≥ 3rd year) students). Results were considered significant at p-values <0.05.

## 3 Results

### 3.1 Response rate and demographics

A total of 351 participants completed the survey. A precise response rate calculation was not feasible due to multiple ways of survey dissemination. Data quality assessment revealed no duplicate entries requiring removal. The mean participant age was 25 years (95 % CI: 23–29). and most participants were from Germany (71 %, n = 251), Switzerland (11 %, n = 39) or Austria (8 %, n = 29). Most respondents were female (58 %, n = 204) and medical students (65 %, n = 229). Among students, 24 % (n = 54) were in preclinical and 76 % (n = 175) in clinical semesters. Demographic details are summarized in Table 1; comparative analyses are presented in Table 2, Table 3.

**Table 1:** Demographic data of all participants.

Variable	Total	Residents	Students	Preclinical	Clinical
**Number**	351	122 (35 %)	229 (65 %)	54 (24 %)	175 (76 %)
**Sex**					
Female	204 (58 %)	70 (57 %)	134 (59 %)	36 (67 %)	98 (56 %)
Male	144 (41 %)	49 (40 %)	95 (42 %)	18 (33 %)	77 (44 %)
Other	3 (1 %)	3 (3 %)	0 (0 %)	0 (0 %)	0 (0 %)
**Age (Years)**	25 (23–29)	30 (28–34)	23 (22–25)	20 (20–22)	24 (23–26)

**Table 2:** Comparison of residents and students‘ аnswers in each category, †Non-normally distributed data presented as median (IQR), compared using Mann-Whitney *U* test; ∗Indicates Chi-Square Test, statistically significant differences (p < 0.05) are shown in **bold**.

Variable	Total	Students	Residents	p-value
**Do you plan a career in neurosurgery?**				**<0.001**∗
Yes	216 (62.1 %)	118 (52.2 %)	98 (80.3 %)	
No	132 (37.9 %)	108 (47.8 %)	24 (19.7 %)	
**Likelihood of selecting a neurosurgical training† (%)**	70 (20–95)	55.5 (19–85)	90 (70–100)	**<0.001**
**What are reasons for choosing neurosurgical training?**				**0.004**∗
Other reason	23 (7.4 %)	15 (7.9 %)	8 (6.8 %)	
Prestige	5 (1.6 %)	4 (2.1 %)	1 (0.9 %)	
Scientific interest	49 (15.9 %)	39 (20.4 %)	10 (8.5 %)	
Fascinating interventions	184 (59.6 %)	111 (58.1 %)	73 (61.9 %)	
Technological progress	7 (2.3 %)	6 (3.1 %)	1 (0.9 %)	
Varied work	36 (11.7 %)	13 (6.8 %)	23 (19.5 %)	
No idea	5 (1.6 %)	3 (1.6 %)	2 (1.7 %)	
**How did you become aware of neurosurgery?**				**0.007**∗
Other	34 (10.1 %)	25 (11.7 %)	9 (7.4 %)	
Social media	26 (7.7 %)	23 (10.8 %)	3 (2.5 %)	
Medical school	133 (39.6 %)	86 (40.2 %)	47 (38.5 %)	
Colleagues	28 (8.3 %)	20 (9.4 %)	8 (6.6 %)	
Dissertation/mentoring	29 (8.6 %)	16 (7.5 %)	13 (10.7 %)	
Internship	86 (25.6 %)	44 (20.6 %)	42 (34.4 %)	
**What are current deterrents from neurosurgery?**				0.082∗
Other	66 (19.1 %)	44 (19.3 %)	22 (18.6 %)	
Antiquated hierarchical structure	57 (16.5 %)	39 (17.1 %)	18 (15.3 %)	
Long working hours	45 (13.0 %)	34 (14.9 %)	11 (9.3 %)	
Inadequate teaching/mentoring	58 (16.8 %)	30 (13.2 %)	28 (23.7 %)	
Too much administrative work	21 (6.1 %)	10 (4.4 %)	11 (9.3 %)	
Too few flexible working concepts	36 (10.4 %)	23 (10.1 %)	13 (11.0 %)	
Training period too long	9 (2.6 %)	7 (3.1 %)	2 (1.7 %)	
24h on-call system	13 (3.8 %)	11 (4.8 %)	2 (1.7 %)	
Limited opportunities for later self-employment	41 (11.8 %)	30 (13.2 %)	11 (9.3 %)	
**What would make the profession more attractive?**				**0.002**∗
Other	24 (7.1 %)	12 (5.5 %)	12 (9.9 %)	
Flexible working models	105 (31.0 %)	80 (36.7 %)	25 (20.7 %)	
Max 12h on-call system	26 (7.7 %)	22 (10.1 %)	4 (3.3 %)	
Fewer administrative tasks	43 (12.7 %)	23 (10.6 %)	20 (16.5 %)	
More practical training opportunities	96 (28.3 %)	54 (24.8 %)	42 (34.7 %)	
Opportunities of a paid research year	29 (8.6 %)	20 (9.2 %)	9 (7.4 %)	
Permanent contracts	16 (4.7 %)	7 (3.2 %)	9 (7.4 %)

**Table 3:** Comparison of students in preclinical and clinical semesters; † Median (IQR), non-parametric comparison using Mann-Whitney *U* test. All shown p-values are **statistically significant** (p < 0.05).

Variable	Preclinical Semesters	Clinical Semesters	p-value
**Number**	54 (23.6 %)	175 (76.4 %)	–
**Sex**			0.164∗
Female	36 (66.7 %)	98 (56.0 %)	
Male	18 (33.3 %)	77 (44.0 %)	
**Age (years)†**	20 (20–22)	24 (23–26)	**<0.001**
**Career in neurosurgery planned**			**0.006**∗
Yes	19 (35.9 %)	99 (57.2 %)	
No	34 (64.2 %)	74 (42.8 %)	
**Likelihood of neurosurgical training† (in %)**	30 (18–59)	62 (20–90)	**0.003**
**Reason for choosing neurosurgery**			0.113∗
Other reason	3 (7.1 %)	12 (8.1 %)	
Prestige	1 (2.4 %)	3 (2.0 %)	
Scientific interest	13 (31.0 %)	26 (17.5 %)	
Fascinating interventions	19 (45.2 %)	92 (61.7 %)	
Technological progress	0 (0.0 %)	6 (4.0 %)	
Varied work	4 (9.5 %)	9 (6.0 %)	
No idea	2 (4.8 %)	1 (0.7 %)	
**How became aware of neurosurgery**			**<0.001**∗
Other	7 (14.3 %)	18 (10.9 %)	
Social media	12 (24.5 %)	11 (6.7 %)	
Medical school	20 (40.8 %)	66 (40.0 %)	
Colleagues	7 (14.3 %)	13 (7.9 %)	
Dissertation/mentoring	0 (0.0 %)	16 (9.7 %)	
Internship	3 (6.1 %)	41 (24.9 %)	
**Current deterrents from neurosurgery**			0.369∗
Other	16 (29.6 %)	28 (16.1 %)	
Antiquated hierarchical structure	11 (20.4 %)	28 (16.1 %)	
Long working hours	5 (9.3 %)	29 (16.7 %)	
Inadequate teaching/mentoring	6 (11.1 %)	24 (13.8 %)	
Too much administrative work	2 (3.7 %)	8 (4.6 %)	
Too few flexible working concepts	5 (9.3 %)	18 (10.3 %)	
Training period too long	0 (0.0 %)	7 (4.0 %)	
24h on-call system	3 (5.6 %)	8 (4.6 %)	
Limited opportunities for later self-employment	6 (11.1 %)	24 (13.8 %)	
**What would make the job more attractive**			0.711∗
Other	2 (4.2 %)	10 (5.9 %)	
Flexible working models	16 (33.3 %)	64 (37.7 %)	
Max 12h on-call system	7 (14.6 %)	15 (8.8 %)	
Fewer administrative tasks	7 (14.6 %)	16 (9.4 %)	
More practical training opportunities	9 (18.8 %)	45 (26.5 %)	
Opportunities of a paid research year	5 (10.4 %)	15 (8.8 %)	
Permanent contracts	2 (4.2 %)	5 (2.9 %)	

### 3.2 Perceived career in neurosurgery

**Fig. 1:**
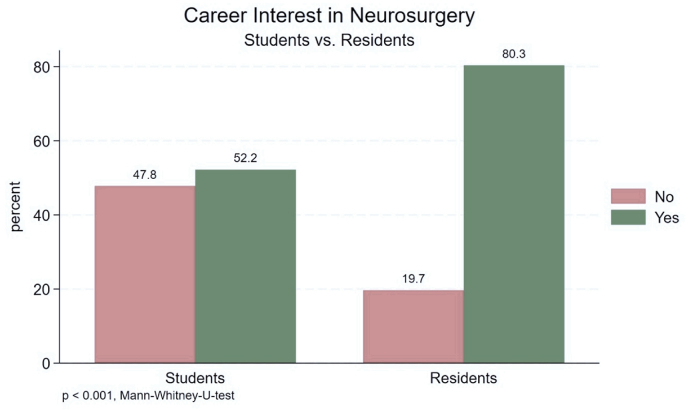
Perceiving career in neurosurgery - Students vs Residents.

**Fig. 2:**
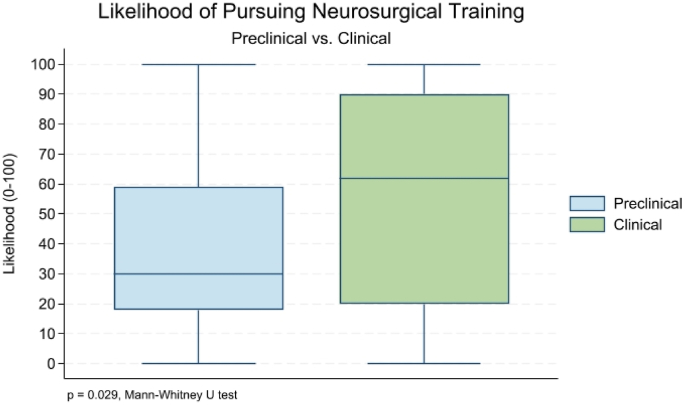
Likelihood of pursuing a neurosurgical career among students in preclinical and clinical years.

A high number of participants stated that they were planning a career in neurosurgery (62 %, n = 216). The likelihood of pursuing neurosurgical training was overall very high (70 %, p = 0.001). Residents (80 %, n = 98) were significantly more interested than students (52 %, n = 118) in a career in neurosurgery (p < 0.001) (Fig. 1). Similarly, students in advanced clinical years (57 %, n = 99) stated significantly higher interest in a neurosurgical career than students in preclinical semesters (36 %, n = 19) (p = 0.006) (Fig. 2). Career motivation analysis revealed that the most frequent reasons for choosing neurosurgery among residents and students were the fascination for surgical procedures (60 %, n = 184), scientific interest (16 %, n = 45), and the diverse spectrum of clinical responsibilites (12 %, n = 36). Students in preclinical years were more intrigued by scientific research (31 %, n = 13) than those in clinical years (18 %, n = 26). Conversely, both students in clinical years (62 %, n = 92) and residents (62 %, n = 73) consistently declared surgical procedures as their primary motivation for choosing neurosurgery. Both residents and students in clinical years stated they became aware of neurosurgery mostly through medical school curricula (39 %, n = 47, and 40 %, n = 66) or internships (34 %, n = 42, and 25 %, n = 41) as their predominant source of neurosurgical exposure. However, students in preclinical years responded that they became aware of neurosurgery mostly through medical school curricula (41 %, n = 20) and SoMe (25 %, n = 12).

### 3.3 Current deterrents and desired improvements

**Fig. 3:**
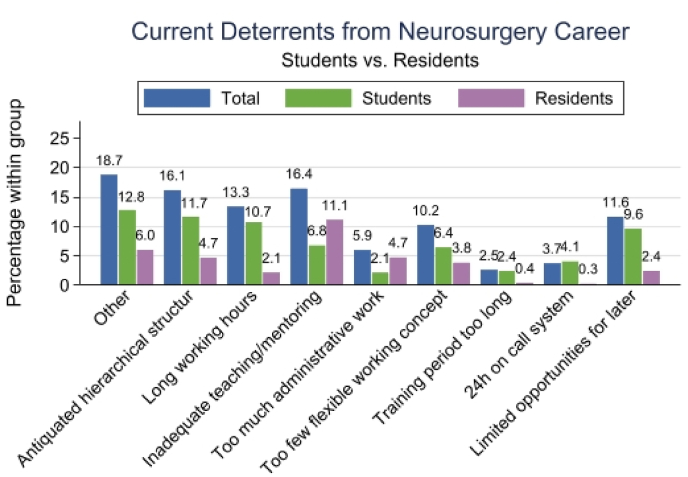
Perceived deterrents to pursuing a career in neurosurgery among medical students and residents.

Participants identified multiple barriers to neurosurgical carrer selection, with the most frequently cited being hierarchical structures (17 %, n = 57), insufficient teaching and mentoring (17 %, n = 58), long working hours (13 %, n = 45), limited possibilities for self-employment (12 %, n = 41), and little workplace flexibility (10 %, n = 36) (Fig. 3). When asked about potential improvements, the majority of participants noted that flexible working models (31 %, n = 105), more practical training opportunities (28 %, n = 96), and fewer administrative tasks (13 %, n = 43) would make a career in neurosurgery more attractive. Notably, options like 12-h on-call shifts (8 %, n = 26) and permanent contracts (5 %, n = 16) were favored only by a few respondents.

**Fig. 4:**
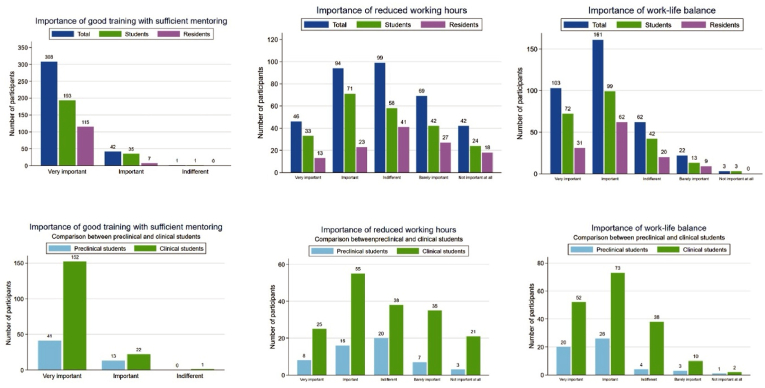
Importance of mentorship, reduced working hours, and work-life balance compared between residents and students and students in preclinical and clinical semesters.

Regarding work-life balance priorities, three-quarters of participants (75 %, n = 264) rated this aspect as very important (29 %, n = 103) or important (46 %, n = 161) (Fig. 4). This aspect appeared to be even more important for students in preclinical years (85 %, n = 46) than in clinical years (71 %, n = 125), albeit not reaching statistical significance (p = 0.209). Structured training was of utmost importance to 99.8 % of all participants (n = 350) (Fig. 4), while rotations at external departments (52 %, n = 183) or abroad (international; 65 %, n = 229) showed more modest interest. Reduced working hours were desired only by 40 % of all participants (n = 140) (Fig. 4), and the use of modern technologies was demanded by 63 % (n = 221) of all respondents.

### 3.4 The role of social media and emerging technologies

Social media usage patterns revealed that 81 % (n = 285) of all participants reported using SoMe for less than 1 h or a maximum of 2 h per day. There was a similar distribution between residents (85 %, n = 104) and students (79 %, n = 181) in SoMe use. However, students in preclinical years indicated spending more time on SoMe, with 50 % (n = 27) using it for up to 2 h and 30 % (n = 16) using it for more than 2 h. The importance of SoMe was moderate for all participants (mean score 44/100 [CI 20–64]), with a higher score among preclinical students (mean 51/100 [CI 33–70]), though this difference did not achieve statistical significance (p = 0.09). Half of all respondents (50 %, n = 174) perceived that neurosurgery is currently underrepresented on SoMe.

Over half of all participants (53 %, n = 186) stated they use artificial intelligence (AI) in their academic or professional daily practice. A higher proportion of students in preclinical years (69 %, n = 37) than in clinical years (52 %, n = 83) declared they use AI either often or very often (p = 0.053). Regarding the use of emerging technologies like Augmented (AR)/Virtual (VR) and Mixed Reality (MR), most respondents (76 %, n = 265) reported minimal or no usage.

## 4 Discussion

### 4.1 Perceptions of a career in neurosurgery

Contrary to expectations of waning interest among Generation Z, a substantial proportion of surveyed participants (62 %, n = 216) expressed a desire to pursue a career in neurosurgery. This inclination was more pronounced among residents (80 %, n = 98) compared to students (52 %, n = 118), a finding that likely reflects the exploratory nature of medical education at the student level, especially during preclinical semesters, when exposure to neurosurgical practice is very limited. However, these findings must be interpreted with considerable caution due to potential selection bias. The high career interest observed may not represent broader Generation Z perspectives, as residents in our survey were already engaged in neurosurgical training, and many of the students were affiliated with neurosurgical societies or interest groups. Consequently, the apparent sustained interest documented in this study may not accurately reflect the broader decline in neurosurgical career attraction that has been reported in the literature. The true extent of decreasing interest among Generation Z medical students may remain underestimated due to our recruitment methodology.

While scientific research possibilities and task diversity were frequently cited as motivation for selecting a neurosurgical training, the primary driver remained the deep fascination with surgical procedures. This finding suggests that the fundamental appeal of neurosurgical procedures continues to resonate with this generation. Awareness and interest in the field typically arise through medical school curricula and clinical internships, as reported by the majority of respondents. This highlights the critical importance of early, high-quality exposure to neurosurgical practice in cultivating career interest.

### 4.2 Deterrents and desired reforms

Career deterrent analysis revealed that participants typically identified multiple interconnected barriers rather than isolated obstacles. Predominant among these was a desire for more flexible working arrangements, notably, not necessarily fewer working hours, which alines with a previous survey conducted among EANS trainees 10 years prior (Stienen et al., 2016b). However, survey data from U.S. neurosurgical residents suggest that duty hour restrictions – mirroring those implemented in European countries since 2003–may adversely affect the quality of residency training (Fargen et al., 2011). Similarly, data from the European Association of Neurosurgical Societies (EANS) indicate a constant international annual decline in the number of procedures performed, supervised, or assisted by residents over recent decades (Stienen et al., 2020). Although simulation-based educational models have been introduced as an alternative (Stengel et al., 2022; Zoia et al., 2022; Hickmann et al., 2022), the traditional hands-on operating room experience remains pivotal (Farah et al., 2023). These training constraints contribute to many residents feeling inadequately prepared to perform certain procedures or to fulfill surgical training requirements, thereby emphasizing the growing demand for more practical training opportunities (Stienen et al., 2016a, 2020).

Centralization of training programs and a reduction in residency sites have been proposed for Germany to address the uneven distribution and diminishing level of expertise among neurosurgeons (Ringel et al., 2023). However, the exclusive nature of neurosurgical training may lose appeal for Generation Z in the face of outdated hierarchies, insufficient mentorship, administrative overload, and rigid work schedules. According to our findings, work-life balance is a pivotal concern for this new generation of medical professionals. Importantly, while only 40 % of participants desired reduced working hours, the majority emphasized the importance of high-quality training supported by modern technologies (Mavrogenis et al., 2024).

Traditionally, neurosurgical residency has been characterized by a high level of intensity, perseverance, and exceptional achievement. Nevertheless, there is a growing need for more balanced and personalized career pathways, including structured mentorship and appropriate workload management. A particularly relevant consideration—though not directly examined in our survey—is the need for parental support and scheduling flexibility, especially given our predominantly female participant cohort (58 %) and persistent gender disparity (Zeitlberger et al., 2022). Although challenging for institutional logistics, similar accommodations have been successfully implemented in specialties such as pediatrics and gynecology, where women are even predominantly employed. In neurosurgery, parental leave remains stigmatized and lacks sufficient infrastructural support despite a continuously growing fraction of female neurosurgeons and rise of shared responsibility concepts (Vayssiere et al., 2023; Stienen et al., 2016c).

Despite these challenges, Germany and Switzerland maintain comparatively high numbers of neurosurgical trainees on both European and global scales (Ringel et al., 2023; Gupta et al., 2024). However, satisfaction levels in prior studies remain average, particularly concerning anatomical and theoretical education and access to simulation or cadaver-based training (Stienen et al., 2016a). Structural reforms in neurosurgical training in both countries are therefore imperative to align with the expectations of Generation Z and optimize career pathway sustainability.

### 4.3 The role of social media and emerging technologies

SoMe has gained significant popularity across different medical specialties, particularly accelerated by the COVID-19 pandemic (Wang et al., 2020). Despite concerns regarding the variability and reliability of content, SoMe platforms offer patients and professionals valuable opportunities for information exchange and collaboration (Shlobin et al., 2021; Abdullah et al., 2024). In the context of neurosurgery, these platforms facilitate global networking and real-time educational engagement. Our survey revealed that 66 % (n = 233) of participants spend at least 1 h daily on SoMe, with 25 % (n = 12) of students reporting that it served as their introduction to neurosurgery. While the overall perceived importance of SoMe was moderate (44/100), its potential to influence academic and professional decisions warrants serious consideration (Waqas et al., 2021).

Interestingly, a recent EANS survey found limited SoMe utilization among European neurosurgical societies and departments – an observation that corresponds to our data, as half of the respondents (50 %, n = 174) felt that neurosurgery is underrepresented on SoMe (Nouri et al., 2022). This might be a significant opportunity to enhance the visibility of the field through platforms such as LinkedIn, X, Meta, and Instagram. Strategic deployment of these channels could be leveraged not only for recruitment and institutional branding but also for educational initiatives (Newall et al., 2021) and the dissemination of clinical and scientific knowledge (Wang et al., 2020).

The survey also indicated remarkably high adoption rates of AI use among participants, with 57 % of students (n = 130) and 46 % of residents (n = 56) reporting regular engagement with AI tools. This pattern mirrors global trends reflecting the rapidly expanding role of machine learning in neurosurgery (Staartjes et al., 2020). Large language models (LLMs), for instance, have demonstrated remarkable accuracy in responding to neurosurgical written board-like questions (Stengel et al., 2024; Guerra et al., 2023), underscoring their value in medical education and decision support. These technologies provide real-time access to extensive medical literature and analytical tools, thereby transforming clinical workflows. Nonetheless, issues such as data security, ethical use, and regulatory compliance must be addressed to ensure responsible integration in neurosurgical training, research, and clinical work (Aamir et al., 2024).

While immersive technologies such as AR/VR/MR present exciting prospects for education (Stengel et al., 2022), their adoption remains limited among current trainees. Greater implementation and research into these tools may help bridge practical training gaps in the future.

## 5 Strengths and limitations

This survey offers valuable insight into the career aspirations and perceptions of Generation Z medical students and residents regarding a future in neurosurgery in Germany and Switzerland. The data may serve as a useful reference for further research and policy development within this field. Our findings provide an initial impression of perceived deterrents to a neurosurgical career among this demographics and identifies potential areas for improvement, findings that align in part with previous studies and may help guide strategic reforms in neurosurgical education and training.

However, several methodological constraints limit the interpretation and generalizability of our findings. Most critically, the reliance on voluntary, non-incentivized participation coupled with subjective self-assessment creates substantial risk of selection bias. This bias is particularly pronounced given our recruitment strategy, which primarily utilized societies and networks, likely attracting participants with pre-existing neurosurgical interests. Consequently, our sample predominantly comprised neurosurgical residents rather than representatives from diverse surgical specialties. Even with broader specialty recruitment, the study's neurosurgical focus would likely have disproportionately attracted neurosurgically oriented participants. Additionally, many students who participated were likely already involved in research projects or PhD programs in neurosurgical departments given our recruitment through specialized associations such as Connectome and professional social media networks. Furthermore, the survey was geographically limited to participants from only two German-speaking countries, which restricts the generalizability of the findings to broader international contexts.

## 6 Conclusion

Despite persistent interest in neurosurgical careers among Generation Z participants in German-speaking countries, traditional barriers—excessive workload, hierarchical structures, and inadequate mentorship—remain significant deterrents. Addressing these challenges through structured mentorship programs, improved practical training opportunities, more flexible working conditions, and stronger institutional support may enhance recruitment and retention in the field. Additionally, the strategic use of SoMe may offer a powerful tool for outreach, education, and fostering global collaboration within the neurosurgical community.

## Declaration of competing interest

The authors declare that they have no known competing financial interests or personal relationships that could have appeared to influence the work reported in this paper.
