# Supplementary material for: Exploring perspectives and boundaries in neurosurgical career pathways for generation Z in German-speaking countries
Source: Brain Spine. 2025 Aug 6;5:104382. doi: 10.1016/j.bas.2025.104382 (PMC12356043; doi:10.1016/j.bas.2025.104382)
Supplement: Multimedia component 1 [file mmc1.docx]

**Gen Z Umfrage – Zukunftsperspektiven in der Neurochirurgie**

1. **Wie alt sind Sie?** (N=? Jahre)
2. **Welches Geschlecht haben Sie?** (Männlich/Weiblich/Divers/N/A)
3. **In welchem Ausbildungsjahr sind Sie?** (N=?)
4. **Möchten Sie eine Karriere in der Neurochirurgie verfolgen?** (Y/N)
5. **Zu wieviel Prozent würden Sie unter den aktuellen Umständen Neurochirurgie als Fachgebiet für Ihre Spezialisierung auswählen? (0-100 %)**
6. **Wenn ja, wieso? (Multiple Choice)**
7. Prestige
8. Wissenschaftliches Interesse
9. Faszinierende Eingriffe
10. Technologische Fortschritte
11. Abwechslungsreiche Tätigkeit
12. Ich weiss es nicht
13. Andere, bitte spezifizieren …..
14. **Wie wurden Sie auf dieses Fachgebiet aufmerksam gemacht? (Multiple Choice)**
15. Soziale Medien
16. Studium
17. Bekannte
18. Mentoren/Doktorarbeit
19. Praktikum (Famulatur, PJ, Krankenpflegepraktikum)
20. Andere, bitte spezifizieren ________

1. **Was schreckt Sie aktuell davon ab Neurochirurgie als Fachgebiet zu machen? (Multiple Choice)**
2. Antiquierte hierarchische Strukturen
3. Lange Arbeitszeiten
4. Unzureichendes Teaching und Mentoring
5. Zu viel administrative Arbeit
6. Zu wenig flexiblen Arbeitzeitkonzepte
7. Zu lange Ausbildungszeit
8. 24 h Schichtsysteme
9. Eingeschränkte Möglichkeiten auf spätere Selbstständigkeit
10. Andere, bitte spezifizieren ______
11. **Was würde den Beruf für Sie attraktiver machen? (Multiple Choice)**
12. Flexible Arbeitsmodele
13. Max. 12 h Dienstmodele
14. Weniger administrative Tätigkeiten
15. Mehr praktische Ausbildungsmöglichkeiten (Workshops, Cadaverlabs, VR/AR)
16. Möglichkeiten eines bezahlten Forschungsjahrs
17. Unbefristete Verträge
18. **Wie wichtig ist für Sie ein ausgewogenes Work-Life Balance mit ausreichend Kompensationszeit?**
19. Sehr wichtig
20. Wichtig
21. Indifferent
22. Kaum wichtig
23. Überhaupt nicht wichtig
24. **Wie wichtig ist für Sie eine gute Ausbildungsmöglichkeit mit ausreichend Teaching/Mentoring?**
25. Sehr wichtig
26. Wichtig
27. Indifferent
28. Kaum wichtig
29. Überhaupt nicht wichtig
30. **Wie wichtig wäre Ihnen die Möglichkeit in Ihrer Ausbildung Rotationen für 1-2 Jahre in Fremdabteilungen (z.B. Neurologie, Neuroradiologie, Orthopädie) zu machen?**
31. Sehr wichtig
32. Wichtig
33. Indifferent
34. Kaum wichtig
35. Gar nicht wichtig
36. **Wie wichtig wäre Ihnen die Möglichkeit in Ihrer Ausbildung Rotationen für 1-2 Jahre in Partnerkliniken im Ausland zu machen (Fellowships)?**
37. Sehr wichtig
38. Wichtig
39. Indifferent
40. Kaum wichtig
41. Gar nicht wichtig
42. **Wie wichtig wäre Ihnen die Möglichkeit ihre Arbeitszeit zu reduzieren mit dem Risiko einer Verlängerung Ihrer Ausbildungszeit?**
43. Sehr wichtig
44. Wichtig
45. Indifferent
46. Kaum wichtig
47. Gar nicht wichtig
48. **Wie wichtig ist Ihnen der Einsatz von modernen Technologien wie Augmented Reality, Künstliche Intelligenz (KI), Robotik?**
49. Sehr wichtig
50. Wichtig
51. Indifferent
52. Kaum wichtig
53. Überhaupt nicht wichtig
54. **Wieviel Zeit verbringen Sie täglich auf sozialen Medien?** (N=? Std)
55. **Wie wichtig sind Ihnen soziale Medien?**
56. Sehr viel
57. Viel
58. Indifferent
59. Kaum
60. Gar nicht
61. **Glauben Sie, dass die Neurochirurgie als Fachgebiet auf sozialen Medien unterrepräsentiert ist bzw würden Sie sich mehr wissenschaftliche Posts von Experten wünschen?**
62. Ja
63. Nein
64. Ich weiß nicht.
65. **Nutzen Sie KI in Ihrem Alltag für private bzw wissenschaftliche Zwecke?**
66. Sehr oft
67. Oft
68. Indifferent
69. Kaum
70. Gar nicht
71. **Nutzen Sie privat, für wissenschaftliche oder Ausbildungszwecke Technologien wie Augmented/Virtual/Mixed Reality (AR/VR/MR)?**
72. Sehr oft
73. Oft
74. Indifferent
75. Kaum
76. Gar nicht

**Gen Z Survey – Future Perspectives in Neurosurgery**

**1) How old are you?** (N = ? years)

**2) What is your gender?** (Male / Female / Diverse / N/A)

**3) What year of training are you in?** (N = ?)

**4) Do you plan to pursue a career in neurosurgery?** (Y/N)

**5) What percentage chance would you currently give to choosing neurosurgery as your specialty?** (0–100%)

**6) If yes, why? (Multiple choice)**

a) Prestige

b) Scientific interest

c) Fascinating surgical procedures

d) Technological advances

e) Diverse range of activities

f) I don’t know

g) Other, please specify: _______

**7) How did you become aware of this specialty? (Multiple choice)**

a) Social media

b) Medical school

c) Friends/Acquaintances

d) Mentors/Thesis supervisor

e) Internship (clinical rotation, final year clerkship, nursing internship)

f) Other, please specify: _______

**8) What currently discourages you from choosing neurosurgery as a specialty? (Multiple choice)**

a) Outdated hierarchical structures

b) Long working hours

c) Inadequate teaching and mentoring

d) Too much administrative work

e) Lack of flexible working time models

f) Training takes too long

g) 24-hour shift systems

h) Limited prospects for future self-employment

i) Other, please specify: _______

**9) What would make the profession more attractive to you? (Multiple choice)**

a) Flexible work models

b) Maximum 12-hour shifts

c) Less administrative work

d) More practical training opportunities (workshops, cadaver labs, VR/AR)

e) Option of a paid research year

f) Permanent employment contracts

**10) How important is a balanced work-life ratio with sufficient time off to you?**

a) Very important

b) Important

c) Indifferent

d) Barely important

e) Not important at all

**11) How important is good training with sufficient teaching and mentoring to you?**

a) Very important

b) Important

c) Indifferent

d) Barely important

e) Not important at all

**12) How important would it be for you to have the option of rotating to other specialties (e.g., neurology, neuroradiology, orthopedics) for 1–2 years during your training?**

a) Very important

b) Important

c) Indifferent

d) Barely important

e) Not important at all

**13) How important would it be for you to have the option of international rotations (fellowships) at partner hospitals abroad for 1–2 years during your training?**

a) Very important

b) Important

c) Indifferent

d) Barely important

e) Not important at all

**14) How important would the option of reducing working hours—with the trade-off of a prolonged training time—be to you?**

a) Very important

b) Important

c) Indifferent

d) Barely important

e) Not important at all

**15) How important is the use of modern technologies such as augmented reality (AR), artificial intelligence (AI), robotics to you?**

a) Very important

b) Important

c) Indifferent

d) Barely important

e) Not important at all

**16) How much time do you spend on social media per day?** (N = ? hours)

**17) How important is social media to you?**

a) Very important

b) Important

c) Indifferent

d) Barely important

e) Not important at all

**18) Do you think neurosurgery is underrepresented on social media, and would you like to see more scientific content from experts?**

a) Yes

b) No

c) I don’t know

**19) Do you use AI in your daily life for personal or scientific purposes?**

a) Very often

b) Often

c) Indifferent

d) Rarely

e) Not at all

**20) Do you use augmented/virtual/mixed reality (AR/VR/MR) technologies for private, scientific, or educational purposes?**

a) Very often

b) Often

c) Indifferent

d) Rarely

e) Not at all
